# Evaluation of the anticancer activity and fatty acids composition of “Handal” (*Citrullus colocynthis* L.) seed oil, a desert plant from south Jordan

**DOI:** 10.1002/fsn3.1994

**Published:** 2020-11-10

**Authors:** Mohammad S. Al‐Hwaiti, Eid M. Alsbou, Ghassan Abu Sheikha, Boulanouar Bakchiche, Thu Huong Pham, Raymond H. Thomas, Sanaa K. Bardaweel

**Affiliations:** ^1^ Department of Environmental Engineering Faculty of Engineering Al‐Hussein Bin Talal University Ma’an Jordan; ^2^ Chemistry Department Faculty of Science Al‐Hussein Bin Talal University Ma’an Jordan; ^3^ Department of Pharmacy Al‐Zaytoonah University of Jordan Ma’an Jordan; ^4^ Laboratory of Process Engineering Faculty of Technology Laghouat University Laghouat Algeria; ^5^ School of Science and the Environment/Boreal Ecosystem Research Facility Grenfell Campus Memorial University of Newfoundland Corner Brook NL Canada; ^6^ Department of Pharmaceutical Sciences School of Pharmacy University of Jordan Amman Jordan

**Keywords:** anticancer, *Citrullus colocynthis*, fatty acids, gas chromatography, hanadal seed oil

## Abstract

**Background:**

The chemical composition of Handal (*Citrullus colocynthis* L.) seed oil cultivated in Jordan deserts was characterized, and its bioactivity was evaluated.

**Methods:**

The oil was extracted from the grinded seeds in 500 ml Soxhlet extractor for 24 hr using n‐hexane, and the recovered fatty acids were methylated with methanolic‐HCL. The fatty acid methyl esters (FAMEs) composition was analyzed using GC‐MS and GC‐FID. The anticancer activity associated with the oil was assessed against colon cancer cell lines (Caco‐2 and HCT‐116) and compared to its cytotoxicity on the human skin fibroblast. Multivariate analysis was used to determine relationship of the fatty acid composition with that of the anticancer activity.

**Results:**

The results demonstrated that fatty acid composition of *Citrullus colocynthis* seed oil chiefly contains Linoleic acid, denoted as C18:2n6 (75%), followed by Palmitic acid C16:0 (8%), Stearic acid C18:0 (5%), and Oleic acid C18:1n9 (9%). It is demonstrated as an excellent source of essential fatty acids omega‐6 (e.g., Linoleic acid), whereas omega‐3 (e.g., α‐Linolenic acid) and hydroxy polyunsaturated fatty acids are found at small level. Interestingly, the oil exhibited reasonable anticancer effects against colorectal cancer cell lines with IC_50_ values varying between 4 and 7 mg/ml. The correlation test revealed a relationship between the fatty acid composition and the effectiveness on treatments.

**Conclusions:**

Handal plant from Jordan appears to have very high level of Linoleic acid compared to other oils measured in different geographic locations and that there appears to be some anticancer activities associated with the fatty acid content of Handal seed oil.

## Graphical abstract

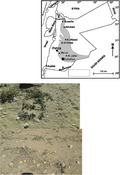

## 1 BACKGROUND

*Citrullus colocynthis* (L.) Schrad is a vegetable plant that is cultivated and geographically dispersed in the desert of Middle East, Asia, North Africa, and Southern Europe (Dane et al., 2006; Hassanane et al., 2001; Rahimi et al., 2012; Rani et al., 2017). Shi et al. (2014) reported that *Citrullus colocynthis* (*C. colocynthis*) plant exhibits a wide range of medicinal uses in leprosy, diabetes, constipation, asthma, bronchitis, jaundice, joint pain, cancer, mastitis, gut disorders, colic, gastroenteritis, dysentery, rheumatism, hypertension, pulmonary, dermatological conditions, and gynecological infections (Aburjai et al., 2007; Delazar et al., 2006; Eddouks et al., 2002; Jayaraman et al., 2009; Kong et al., 2010; Meena & Patni, 2008; Mohammed et al., 2010; Najafi et al., 2010; Nmila et al., 2000; Seger et al., 2005).

Medicinal plants are currently of great interest as natural sources for new anticancer agents due to their potent antioxidant capacity, antimutagenic properties, low side effects, low cost, and being easily accessible (Chekroun et al., 2015; De Martel et al., 2012; Hussain et al., 2014; Kim et al., 2015; Shokrzadeh et al., 2010). Phytochemical studies have shown that *Citrullus colocynthis* (L.) Schrad plant is a rich source of flavonoids and alkaloids with proven anticancer activities (Patal & Krishnamurthy, 2013).

The composition of the *Citrullus colocynthis* seed oil from various areas in the world was reported to contain 13.19%–26.86% protein, 14.48%–24.62% fat, and 2.00%–4.46% ash (Cantarelli et al., 1993; Cristina et al., 2012; Hassimi & Claude, 2007; Lazos & Kalathenos, 1988). It has been reported that fatty acid composition (%) of *Citrullus colocynthis* in seed oil ranged between 67% and 73% for linoleic acid, 10 and 16% for oleic acid, 5 and 8% for stearic acid, and 9 and 12% for palmitic acid (Gurudeeban et al., 2010; Nikolaos & Theophanis, 2000; Schafferman et al., 1998).

Vegetable oils are known as natural products that contain fatty acids of different saturation levels (Nesma et al., 2010). They also contain triglycerides, diglycerides, monoglycerides, and vitamins (A, D, E, and K) in small amounts (Emmanuel & Mudiakeoghene, 2008). Nonetheless, fatty acids are often classified into long‐chain fatty acids, with more than 14 carbon atoms and higher melting points, and short‐chain fatty acids with lower than six carbon atoms and lower melting points (Fasina et al., 2006).

In Jordan, *Citrullus colocynthis* (Handal) is creeping plant growing in arid deserts located in southern and eastern parts of Jordan. Handal crops cost harvesting and extracting are highly economic relative to other vegetable oils. The present study aims to characterize the chemical composition of fatty acids in Handal seed oil cultivated in Jordan deserts and assess the potential use of this oil as anticancer agent against colon cancer cells.

## 2 METHODS

### 2.1 Seed material

**Figure 1:**
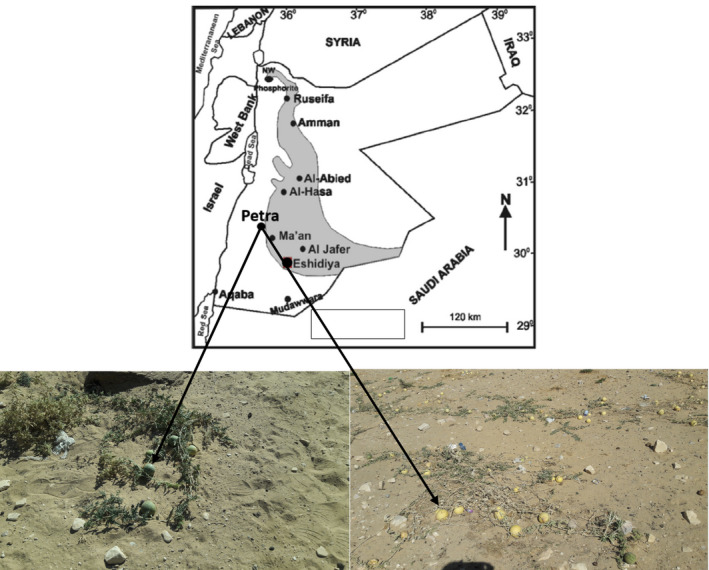
Location map showing the Handal fruits distribution in south Jordan (adopted from Al‐Hwaiti et al. 2015). ***Al‐Hwaiti, M.,*** *Araf, K, Harara, M. (* ***2015*** *). Removal of heavy metals from waste phosphogypsum materials using polyethylene glycol and polyvinyl alcohol methods. Arabian Journal of chemistry, 12, 3141–3150*

Handal fruits were collected from desert areas in south Jordan (Figure 1) over a number of growing seasons. The plant material was identified by a botanist from The University of Jordan and deposited with a voucher specimen (ID: C.CorHud‐04–2018) in the Department of Pharmaceutical Sciences, School of Pharmacy, UJ. Seeds were deshelled and dried at room temperature overnight. The dried Handal seeds were ground using an electric blender. Seed oil was then extracted at 25°C using a Soxhlet extractor to determine total fat content. No approval/permission to collect the plant/fruit samples was required.

### 2.2 Oil extraction

The oil was extracted from the grinded seeds in 500 ml Soxhlet extraction for 24 hr using n‐hexane. The extract was filtered and transferred to a rotary evaporator (LabTech, Germany) to remove n‐hexane under vacuum at 40°C. The raw oil was cloudy yellow color and contained some suspected impurities, such as water, and coextracted compounds. Therefore, it was refined by a set of pretreatment processes. These processes included adding silica gel (5 g per 100 ml oil) to the oil to remove any coagulated and gummy compounds. The mixture (oil and silica gel) was stored at 4°C for 120 min and then centrifuged at 3,500 rpm for 5 min. The oil layer was poured into separatory funnel and washed with distilled water (5 ml water:100 ml oil) for 15 min. After that, the oil was heated to 105°C for 30 min to remove any water and residues. Finally, the oil was bubbled (dried) with a stream of N_2_‐gas (grad 5). The refined oil was then stored for physical, chemical, and biological analysis.

### 2.3 Fatty acids analysis using GC/MS and GC/FID

Handal seed oil fatty acids were esterified to fatty acid methyl esters (FAMEs). A 300 μl of the seed oil was spiked with internal standard (30 μl of C18:0 alkane, 1 mg/ml in n‐hexane). Esterification process was performed using 500 μl of 1.5N methanolic‐HCl (Sigma‐Aldrich). Then, the content was vortexed and incubated (60°C) for 30 min. Following incubation, distilled water (0.8 ml) was added to the cooled samples, and the FAMEs extracted 3 times with 500 μl hexane. The combined fractions were dried under N_2_ and resuspended in n‐hexane (300 μl).

The FAMEs composition was analyzed using GC‐MS and GC‐FID. GC‐MS analysis of Handal seed oil fatty acids was performed using a Trace 1,300 gas chromatography (GC) coupled to a TSQ 8,000 Triple Quadrupole mass spectrometer (ThermoScientific). Methylated fatty acids were separated using a BPX70 high resolution column (10 m × 0.1 mm ID × 0.2 μm) (Canadian Life Science, ON, Canada) using helium as the carrier gas at a flow rate of 1 ml/min. A split mode (1:15) injection was applied with 1 μL volume injection of each sample using a Tri‐plus auto‐sampler (Thermo Scientific). The oven temperature program was set as follow: the initial oven temperature of 50°C (held for 0.75 min), then programmed to increase at 40°C/min to 155°C, then increased at 6°C/min to 210°C, and then increased at 15°C/min to 250°C (held for 2 min), total time: 17 min.

GC‐FID analysis of Handal seed oil fatty acids was conducted using a Trace 1,300 gas chromatography coupled to a Flame Ionization Detector (Thermo Fisher Scientific). Methylated fatty acids were separated based on the same GC parameters as described above for GC‐MS analysis.

The identification of the methylated fatty acids was accomplished by mass spectrum elucidation (for GC‐MS), and comparison of the retention times (for GC‐MS and GC‐FID) and mass spectra to those of the commercial standards (for GC‐MS) (Supelco FAME mix C8–C24, Supelco 37 component mix, Supelco PUFA No. 3; Sigma Aldrich). C18:0 alkane was employed as internal standard. Standard curves were employed in the two analysis methods to determine the amount of individual fatty acids in Handal seed oil, and values are presented as weight %.

### 2.4 Anticancer activity

Normal skin fibroblasts were cultured in DMEM/F12 (Dulbecco's modified essential medium/Ham's 12 nutrient mixture, Gibco). Colorectal cancer cell lines, Caco‐2 and HCT‐116, were cultured in DMEM medium (Dulbecco's Modified Eagle's Medium). Both media were enriched with 10% Fetal Bovine Serum, 100 U/ml of Penicillin, and 100 µg/ml of Streptomycin. Cells were maintained at 37ºC in a humidified 5% CO_2_ incubator. Cell viability was determined by vital staining with trypan blue (0.4% (w/v); Sigma), and cells were counted using a light microscope (Bardaweel et al., 2015).

In vitro assessment of the oil antiproliferative activity was carried out using the Promega CellTiter 96^®^ AQueous Non‐Radioactive Cell Proliferation (MTS) assay to evaluate the number of viable cells in media (Promega 2005). The oil was applied to test wells at concentrations of 5, 10, 20, and 50 mg/ml and incubated at 37ºC with 5% CO_2_ for exposure period of 48 hr. After the completion of the incubation time, the MTS mixture (20 μl/well) was employed. A microplate enzyme‐linked immuno‐assay (ELISA) reader was utilized to read absorbance of the formazan product at 492 nm. Each point was performed in triplicates (Bardaweel et al., 2015).

### 2.5 Statistical analysis

The chemical parameters measured included lipid analysis of the seed oil, and IC_50_ against the cancer cell lines. All measurements were made in four replications. Effects of the treatments on the chemical parameters were done using one‐way analysis of variance (ANOVA) and means separated using Fisher's LSD at α = 0.05. Compositional distributions of the fatty acids in Handel seed oil are done using pie charts. Principal component analysis was used to discern relationship between the fatty acid composition of the seed oil and anticancer activity in the cancer cell lines. XLSTATS premium version (Addinsoft) was used for the multivariate analysis. Statistical analysis was performed applying the Student *t* test using SPSS 10.0 statistical software package (SPSSFW, SPSS Inc). A *p* value < .05 was considered statistically significant for the cell lines treated with different concentrations of the oil.

## 3 RESULTS AND DISCUSSION

**Figure 2:**
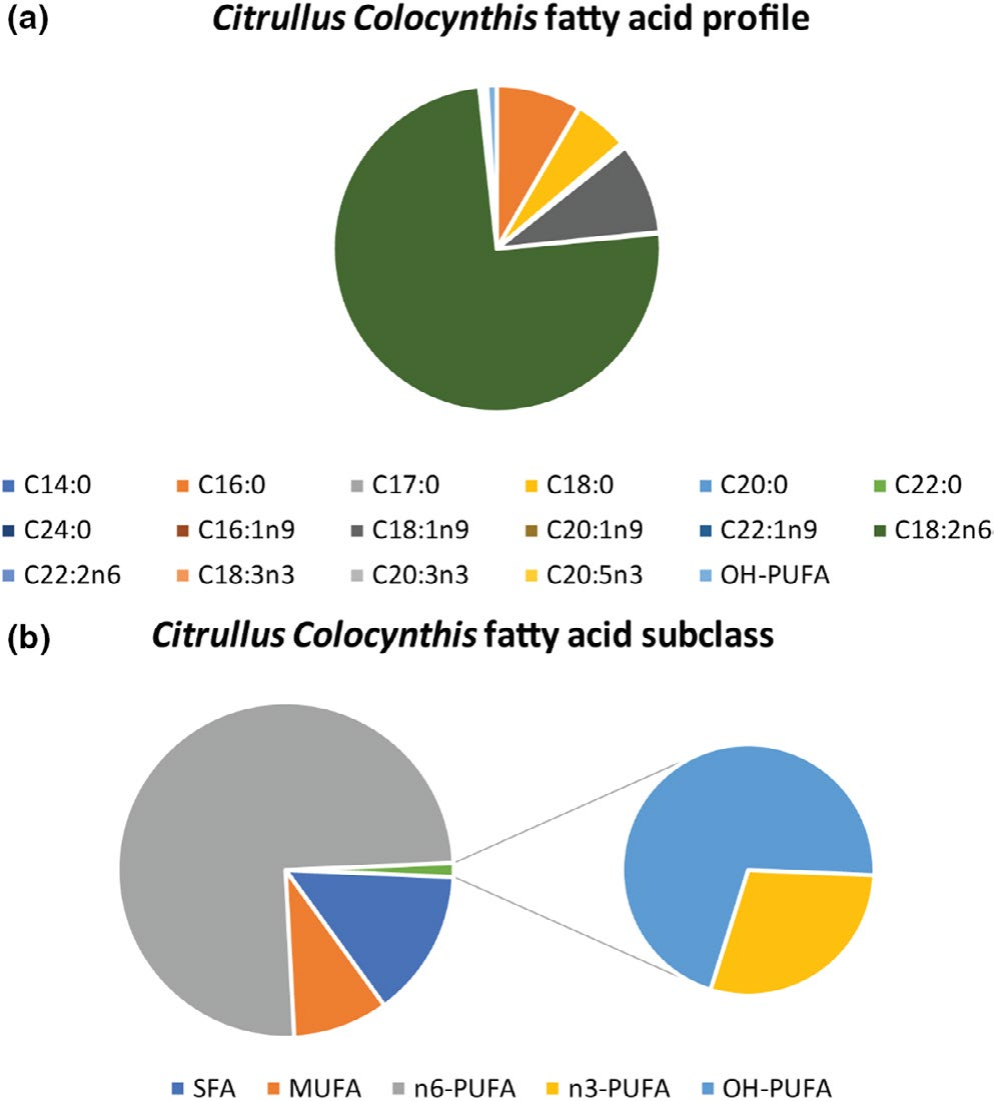
(a) The fatty acid distribution of Handal (*Citrullus Colocynthis*) seeds oil cultivated in Jordan and (b) The distribution of fatty acid subclasses in the oil. SFA = Saturated fatty acids, MUFA = monounsaturated fatty acids, n6‐PUFA = omega 6 polyunsaturated fatty acids, n3‐PUFA = omega 3 polyunsaturated fatty acids, OH‐PUFA = polyunsaturated hydroxy fatty acids. Values represent means, and four replicates were done for each treatment

Fatty acids composition of Handal (*Citrullus colocynthis*) seeds oil is demonstrated in Table 1 and Figure 2. The fatty acid profile of Handal seed oil from Jordan desert is very rich in omega 6‐ polyunsaturated fatty acids, denoted as n6‐PUFA reaching a value of 75.15% by weight (Table 1). The highest contribution to n6‐PUFA is linoleic acid accounting for 74.77% of the total fatty acids (Figure 2). Saturated fatty acids in Handal oil made up 14.33% of the oil and are mainly composed of palmitic acid (C16:0) and stearic acid (C18:0). Total monounsaturated fatty acids (MUFA) were 9.18% in which oleic acid (C18:1n9) is the major component as monounsaturated. Omega 3‐ and hydroxy polyunsaturated fatty acids are found at minor levels, that is, n3‐PUFA and OH‐PUFA were shown as 0.39% and 0.94%, respectively, in Table 1.

**Table 1:** Fatty acids composition of Handal (*Citrullus Colocynthis*) seeds oil

MW	FAME	%wt	MW	FAME	%wt	Subclass	%wt
242	C14:0	0.017 ± 0.003	296	C18:1n9	9.04 ± 0.09	SFA	14.33 ± 0.02
270	C16:0	8.35 ± 0.03	324	C20:1n9	0.083 ± 0.006	MUFA	9.18 ± 0.03
284	C17:0	0.075 ± 0.004	352	C22:1n9	0.050 ± 0.003	n6‐PUFA	75.15 ± 0.04
298	C18:0	5.36 ± 0.06	294	C18:2n6	74.8 ± 0.1	n3‐PUFA	0.389 ± 0.006
326	C20:0	0.177 ± 0.007	350	C22:2n6	0.38 ± 0.02	OH‐PUFA	0.94 ± 0.01
354	C22:0	0.12 ± 0.01	292	C18:3n3	0.099 ± 0.004	Total (%)	100
382	C24:0	0.24 ± 0.01	320	C20:3n3	0.03 ± 0.01		
268	C16:1n9	0.008 ± 0.002	316	C20:5n3	0.26 ± 0.01		

Note

Values (% by weight composition) represent means ± standard errors. The fatty acid was detected in form of fatty acid methyl ester (FAME). The components with the C number before the colon represent total number of carbons, while the numbers after the colon represent the total number of double bonds, *n*‐ represent the position of the first double bond counting from the methyl end or omega end (e.g., C18:3n3 = omega 3‐ linolenic acid or α‐linolenic acid).

Abbreviation: SFA, Saturated fatty acids; MUFA, monounsaturated fatty acids; n6‐PUFA, omega 6 polyunsaturated fatty acids; n3‐PUFA, omega 3 polyunsaturated fatty acids; OH‐PUFA, polyunsaturated hydroxy fatty acids.

The results from the current study are in close agreement with previous literature reports on fatty acids composition of *Citrullus colocynthis* L. seed oil from different origins (Ashish et al., 2010; Kamalakar et al., 2015; Kulkarni et al., 2012; Solomon et al., 2010; Zohara et al., 1999) (Table 2). It was found that seed oil is composed of four major fatty acids: palmitic, stearic, oleic, and linoleic acids. Of the four major fatty acids, linoleic acid is the most prevalent with the value ranging between 55.90% and 74.77%, with the most prominent level found in Jordan seed oil (74.77%). Palmitic acid (C16:0) ranged from 8.35% in oil from Jordan and compared to 11.70% in oil from Nagpur and was the predominant saturated fatty acid. The composition of total saturated fatty acids (Palmitic C16:0 and Stearic C18:0) and unsaturated fatty acid (Oleic and Linoleic) contents of the oil was reported between 13.71–21.40% and 77.50–83.81%, respectively (Table 2). Due to the different origin of the *Citrullus colocynthis* seed and an effect of laboratory variation, the lowest saturated fatty acids were seen from the oil produced in Jordan (this study) and the highest were reported from the group in Nagpur, India. In contrast, Jordan's Handal oil contains the highest unsaturated content (83.81%) as compared to the lowest (77.5%) in the oil obtained from Nagpur (Kulkarni et al., 2012).

**Table 2:** Fatty acid composition (by wt%) of *Citrullus colocynthis* L. seed oil in Jordan compared to other countries

	India^a^	India^b^	India^c^	India^d^	Malaysia^e^	Israel^f^	Jordan
Fatty acids	wt %	wt %	wt %	wt %	wt %	wt %	wt %
Palmitic acid (C_15_H_31_COOH)	9.38	10.30	11.70	10.43	10.48	10.10	8.35
Stearic acid (C_17_H_35_COOH)	7.34	8.00	9.70	9.84	9.72	6.70	5.36
Oleic acid (C_17_H_33_COOH)	17.04	24.50	11.40	15.90	17.95	13.10	9.04
Linoleic acid (C_17_H_31_COOH)	61.05	55.9	66.10	62.81	61.41	70.10	74.77

^a^ Ashish et al. (2010).

^b^ Kamalakar et al. (2015).

^c^ Kulkarni et al. (2012).

^d^ Gurudeeban et al. (2010).

^e^ Solomon et al. (2010).

^f^ Zohara et al. (1999).

### 3.1 Cytotoxicity

Cell growth of cancer cells (Caco‐2 and HCT‐116) and normal skin fibroblast cells were assessed with the MTS assay after 48h exposure period. The IC_50_ values, described as the concentration at which 50% of cell growth is inhibited, are presented in Table 3. Notably, there was statistically significant difference between cancer cell growth in wells treated with the examined oil, relative to the growth of normal skin fibroblasts treated with same concentration of the oil. The results indicated that cancer cell viability was considerably affected after 48 hr exposure relative to the human fibroblasts upon treatment with the examined oil, using the MTS assay at concentration points up to 50 mg/ml, suggesting a reasonable anticancer activity and safety profile against the human skin fibroblasts (Bardaweel et al., 2013). However, health complications such as colic, diarrhea, vomiting, and liver impairment have been frequently reported with the use of *C. colocynthis* (Jouad et al., 2001).

**Table 3:** The IC_50_ values (mg/ml) of the oil on three human cancer cell lines

Cell line	Caco−2	HCT−116	Fibroblasts
Oil	7 ± 0.9*	4 ± 0.3*	88 ± 6*

Values are expressed as mean ± *SD* (*n* = 9).

* *p* < .05.

### 3.2 Relationship between anticancer activity and *Citrullus colocynthis* L. seed oil fatty acid composition

**Figure 3:**
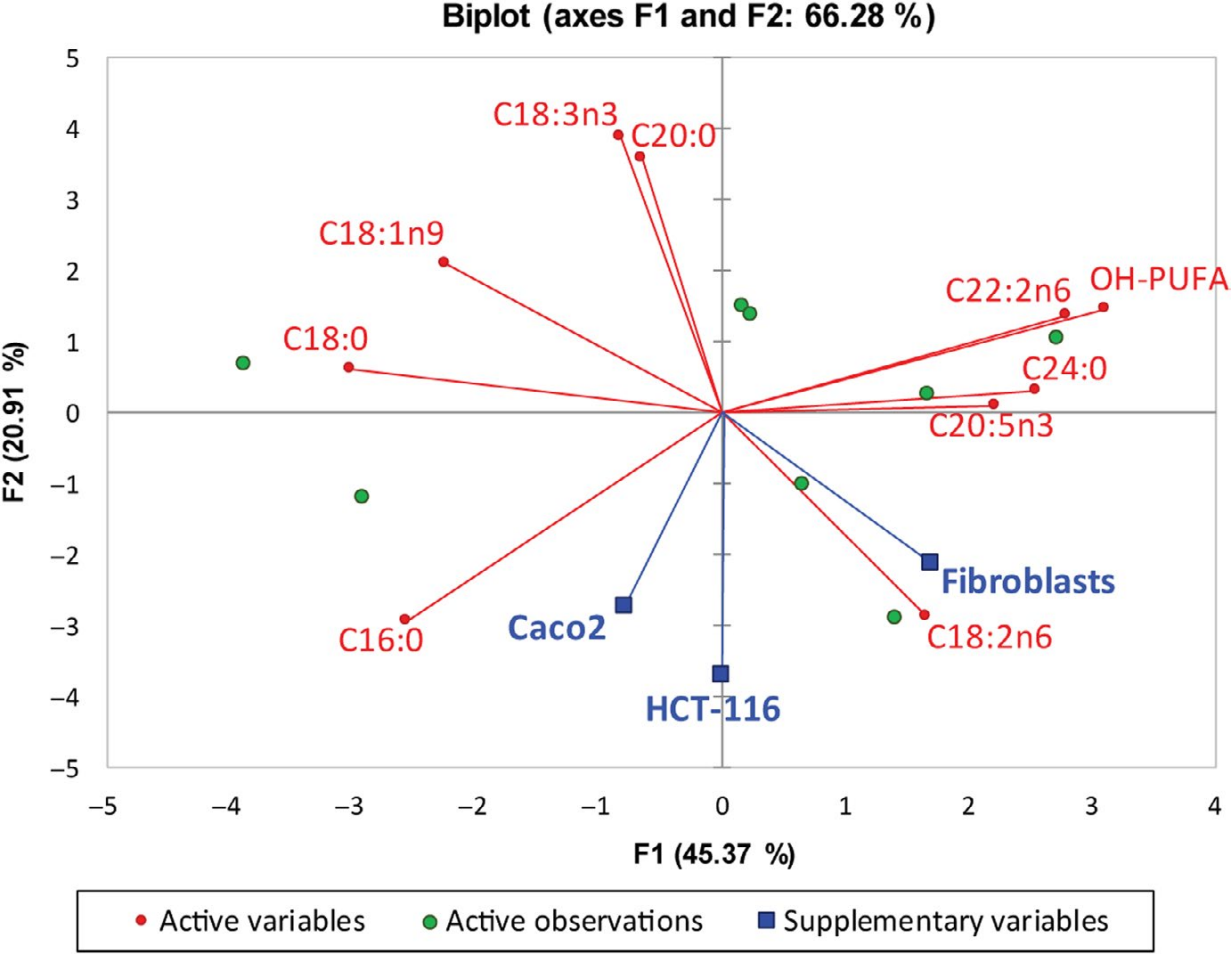
Biplot showing relationships between the fatty acid composition of *Citrullus colocynthis* L. seed oil from Jordan and suppression of cellular growth (IC_50_) in 2 cancer cell lines and normal skin fibroblasts

Principal component analysis (PCA) was then employed to determine whether the functional fatty acids in the Handal oil are in correlation with the cell line treatment efficiency. The segregation of major fatty acids and the cell lines treated with the examined oil into different quadrants of the biplot (Figure 3) showing their potential correlation. The level of Palmitic acid C16:0 was in close correlation circle with the grow inhibition of Caco‐2 cancer cells, while the high concentration of Linoleic acid C18:2n6 in Handal oil was clustered with the growth of normal skin fibroblasts. The HCT‐116 cancer cell line treatment was shown to have significant correlation with the levels of α‐Linolenic acid C18:3n3 and Arachidic acid C20:0 (value in bold shown in Table 4).

**Table 4:** Squared cosines of the variables showing the correlations of fatty acid composition and the three cell lines treatment efficiency. (move Table S‐1 to Supplementary)

	F1	F2	F3	F4	F5	F6	F7
C16:0	**0.557**	0.333	0.034	0.061	0.006	0.001	0.009
C18:0	**0.769**	0.015	0.055	0.069	0.077	0.012	0.003
C20:0	0.058	**0.587**	0.185	0.000	0.150	0.019	0.002
C24:0	**0.555**	0.004	0.333	0.087	0.000	0.021	0.001
C18:1n9	**0.426**	0.173	0.188	0.203	0.006	0.003	0.000
C18:2n6	0.233	0.324	**0.423**	0.010	0.010	0.000	0.000
C22:2n6	**0.665**	0.072	0.007	0.149	0.099	0.000	0.009
C18:3n3	0.035	**0.499**	0.071	0.344	0.033	0.016	0.001
C20:5n3	**0.420**	0.000	0.316	0.097	0.158	0.008	0.000
OH‐PUFA	**0.820**	0.084	0.007	0.007	0.078	0.001	0.004
Caco2	0.053	0.286	0.137	0.019	**0.427**	0.001	0.077
HCT−116	0.000	**0.534**	0.012	0.014	0.377	0.034	0.029
Fibroblasts	0.243	0.173	**0.307**	0.002	0.006	0.219	0.051

Note

The results corresponding to the supplementary variables are displayed in the second part of the table.

Values in bold correspond for each variable to the factor for which the squared cosine is the largest.

## 4 CONCLUSIONS

The major fatty acids present in Handal seed oil from the desert of Jordan are Linoleic acid 74.8 ± 0.1 (%), Palmitic acid 8.35 ± 0.03 (%), Stearic acid 5.36 ± 0.06 (%), and Oleic acid 9.04 ± 0.09 (%), while Omega 3‐ and hydroxy polyunsaturated fatty acids are found at less than 1%. The content of linoleic oil in this study is higher than that of Handel oil from other regions. The results of the present study concluded that cancer cell viability was considerably affected after 48 hr exposure relative to the human fibroblasts upon treatment with the examined oil, suggesting a reasonable anticancer activity and safety profile against the human skin fibroblasts. The principal component analysis (PCA) suggests a potential relationship between the high omega‐6 fatty acid in natural Handal seed oil and the anticancer activities reported against the studied cancer cell lines.

Overall, these findings indicate that Handal plant from Jordan has very high level of linoleic acid compared to other oils measured in different geographic locations and that there appears to be some anticancer activities associated with the fatty acid profile of Handel seed oil. Further studies using isolated constituents instead of the whole extract should be carried out to better understand the relationship between Handel seed oil composition and potential anticancer activity.

## CONFLICTS OF INTERESTS

Not applicable.

## AUTHORS’ CONTRIBUTIONS

MA, RT, and SB conceived and designed the experiments; EA, GA, BB, and TP performed the experiments; TP, SB, RT, and MA analyzed the data; MA, SB, AT, and TP wrote the paper; MA and SB supervised the project. All authors read and approved.

## ETHICAL APPROVAL

Not applicable.

## CONSENT FOR PUBLICATION

Not applicable.

## Data Availability

The datasets used and/or analyzed in the current study are available from the corresponding author on reasonable request.
